# Microstructure Evolution during Hot Deformation of UNS S32750 Super-Duplex Stainless Steel Alloy

**DOI:** 10.3390/ma14143916

**Published:** 2021-07-14

**Authors:** Elisabeta Mirela Cojocaru, Anna Nocivin, Doina Răducanu, Mariana Lucia Angelescu, Ion Cinca, Irina Varvara Balkan, Nicolae Șerban, Vasile Dănuț Cojocaru

**Affiliations:** 1 Materials Science and Engineering Faculty, University POLITEHNICA of Bucharest, 060042 Bucharest, Romania; mirela.cojocaru@mdef.pub.ro (E.M.C.); doina.raducanu@upb.ro (D.R.); lucia.angelescu@mdef.pub.ro (M.L.A.); ion.cinca@upb.ro (I.C.); irina.balkan@mdef.pub.ro (I.V.B.); nicolae.serban@upb.ro (N.Ș.); 2 Faculty of Mechanical, Industrial and Maritime Engineering, Ovidius University of Constanța, 900527 Constanța, Romania; anocivin@univ-ovidius.ro

**Keywords:** super-duplex stainless steel (SDSS), SEM-EBSD microstructural analysis

## Abstract

The present paper analyzes UNS S32750 Super-Duplex Stainless Steel hot deformation behavior during processing by upsetting. The objective of this paper is to determine the optimum range of deformation temperatures, considering that both austenite and ferrite have different deformation behaviors due to their different morphology, physical, and mechanical properties. Because the capability of plastic deformation accommodation of ferrite is reduced when compared to austenite, side cracks and fissures can form during the hot deformation process. Consequently, it is important to find the optimum conditions of deformation of this type of stainless steel to establish the best processing parameters without deteriorating the material. The experimental program involved the application of hot deformation by the upsetting method on a series of samples between 1000 °C and 1275 °C, with a total degree of deformation of 30%. The resultant samples were examined by SEM-EBSD to establish and analyze the evolution of the phases present in the structure from several points of view: nature, distribution, morphology (size and shape), and their structural homogeneity. The GROD (Grain Reference Orientation Deviation) distribution map was also determined while taking into account the possible precipitation of the secondary austenite phase (γ_2_-phase) and the analysis of the dynamic recrystallization process according to the applied deformation temperature. The main conclusion was that UNS S32750 SDSS steel can be safely deformed by upsetting between 1050–1275 °C, with an experimented total degree of deformation of 30%.

## 1. Introduction

The material considered in this experimental research is Super-Duplex Stainless Steel (SDSS), which shows a bi-phasic microstructure, composed of proportionally equal phases of, δ ferrite, and γ austenite, with a sufficient content of Cr, Mo, and N to deliver high corrosion resistance to pitting and to stress corrosion cracking [1,2]. In addition to these important characteristics, SDSS displays an industrially recognized combination of high mechanical strength and toughness [3,4]. However, this stainless steel represents only approximately 1% of the total production of stainless steels manufactured, including other two important types, austenitic stainless steels (ASSs), and ferritic stainless steels (FSSs) [5,6]; compared to these two types of steel, one problem arises for SDSS due to the distinct mechanical behavior of the two contenting phases that can lead to non-uniform deformation in the case of thermomechanical processing of this material. It is known that the main thermomechanical processing parameters (deformation temperature and applied deformation degree) play a crucial role in all the hot-deformation processes of metallic materials. This is because the correct selection and control of these parameters can prevent the unwanted generation of defects in the final products [7,8,9,10]. A suitable hot-deformation regime must be established according to the distinct properties of the contenting phases of the metallic material, such as: phase morphology, flow stress, strain hardening coefficient, etc. In the case of SDSS, both stresses and strains from the applied hot working regime are unevenly distributed in ferrite δ and austenite γ due to the lower capability of plastic deformation accommodation of ferrite than that of austenite. This triggers different deformation behavior for degrees of deformation [11,12,13,14,15]. It has been reported that, in the case of applied recrystallization treatments prior to hot deformation processing, the initial grains of γ austenite are smaller than the grains of δ ferrite, with a direct repercussion on the microstructure of the alloy that was obtained after hot deformation, resulting in a more homogenous deformed γ austenite phase compared to the δ ferrite phase [16,17]. In addition, several reports indicate that micro-cracks may appear in the δ phase, which expand towards phase boundaries, leading to sample failure [15,18,19,20,21,22]. It stands to reason that finding and establishing the hot deformation temperature and the applied deformation degree are important goals for the optimal thermomechanical processing of SDSS alloys. Consequently, the main objectives of this research were the determination of the optimum hot deformation temperatures range of the UNS S32750 SDSS alloy, and the study of the main microstructural changes occurring during hot deformation. The optimal degree of deformation for the UNS S32750 SDSS alloy, was analyzed in a previous paper [23]. The current paper represents a continuation of these earlier experiments, with the aim of establishing a whole package of optimal, useful, and necessary hot deformation processing parameters.

## 2. Materials and Methods

### 2.1. Thermomechanical Processing Route

From the as-received (AR) UNS S32750 SDSS alloy (Sverdrup Steel, Stavanger, Norway), cylindrical-shaped samples with an h/d ratio of 1.5 (height h = 27 mm, and diameter d = 18 mm) were machined. The inspection with penetrant liquid was used to verify, porosity, presence of microcracks, laps (pre-existing defects), etc., on the lateral surface of all samples. Hot deformation of samples was conducted by upsetting, in axial compression, up to 30% total deformation degrees, by applying a 0.37 s^−1^ strain rate (a crosshead speed of 10 mm/s, consistently maintained). The deformation temperature range was selected between 1000 °C and 1275 °C, in 25 °C steps. For “freezing” the internal microstructure after the hot deformation processing, all samples were cooled in water. The inspection with penetrant liquid was used after cooling to again investigate the samples for fissures/microcracks. A detailed presentation of the thermomechanical processing equipment used is presented in an earlier paper [23].

### 2.2. Microstructural Characterization

**Figure 1:**
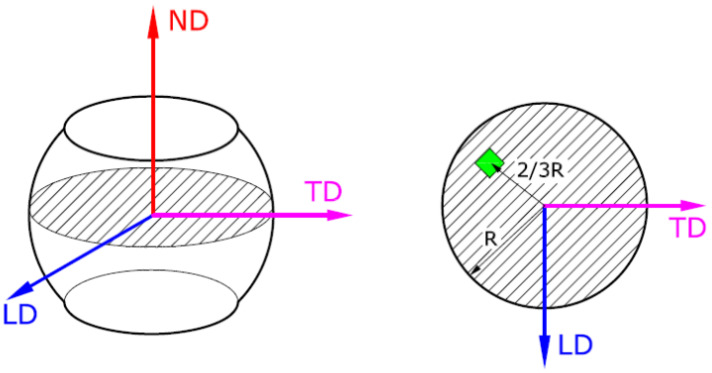
The reference system for the investigated samples: the initial deformed sample (**left**); the LD-TD section at middle-height of sample (**right**); the area investigated by SEM-EBSD analysis (green square); longitudinal direction (LD); transverse direction (TD); normal direction (ND).

The reference system of the samples is shown schematically in Figure 1. Considering this system, the samples were investigated in the LD-TD plane, in a/the selected area, situated two thirds (2/3) from the sample center. For cutting the samples, a precision Metkon MICRACUT 200 (Metkon Instruments Inc., Bursa, Turkey) diamond cutting equipment was used. All samples were hot mounted in conductive phenolic resin (NX-MET, Echirolles, France) at 138 °C and 10 min holding time. The mounted samples were further polished, using a Metkon Digiprep ACCURA (Metkon Instruments Inc., Bursa, Turkey) machine. An additional super-polishing phase was conducted on a Buehler VibroMet™ 2 machine (Buehler, Lake Bluff, IL, USA) for improving the sample surface quality. The polishing and super-polishing phases of sample preparation are presented in detail in a previous paper [23].

A TESCAN VEGA II—XMU (TESCAN, Brno, Czech Republic) scanning electron microscope (SEM) was used for the microstructural analysis using the SEM-EBSD technique. This microscope is equipped with an EBSD detector—BRUKER Quantax eFlash (Bruker Corporation, Billerica, MA, USA). According to Figure 1, the analysis was conducted on the LD-TD plane, at middle height, and a distance from the sample axis equal to 2R/3 (R is the radius of the sample in the LD-TD plane). The phases considered for identifying the microstructural constituents the of investigated UNS S32750 SDSS alloy, were as follows: austenite phase (γ) and ferrite phase (δ). Both phases were indexed in the cubic system (γ-225 and δ-229), the space group Fm3m for γ and Im3m for δ, and the lattice parameter a = 3.66 Å for γ and a = 2.86 Å for δ, respectively. The parameters applied for the SEM-EBSD analysis were as follows: a magnification of × 300, a resolution of 320 pixels × 240 pixels, an acquisition time per pixel of 10 ms, a binning size of 1 × 1, and zero solutions below 3%.

For the constituent phases, the weight fraction (proportion), the nature, morphology, distribution, grain size, structural homogeneity, and dynamic recrystallization, were analyzed in correlation with the considered hot upsetting temperature.

## 3. Results and Discussion

### 3.1. The As-Received (AR) UNS S32750 Super-Duplex Stainless Steel

**Figure 2:**
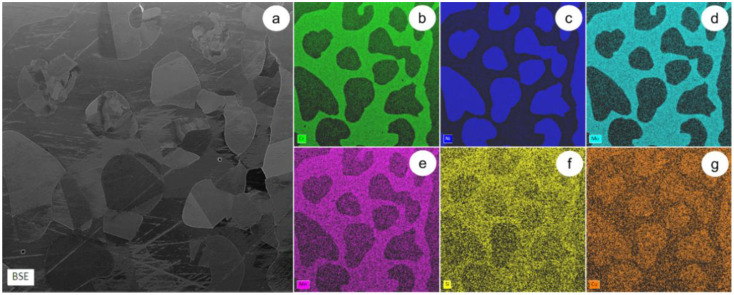
SEM-EDS colorized maps showing the main alloying elements distribution in the AR UNS S32750 Super-Duplex Stainless Steel: SEM-BSE image of the microstructure (**a**); distribution map of Cr (**b**); Ni (**c**); Mo (**d**); Mn (**e**); Si (**f**); Cu (**g**).

The SEM-EDS technique was used to investigate the chemical composition of as-received (AR) UNS S32750 Super-Duplex Stainless Steel. Figure 2a shows a representative SEM-BSE image for the investigated SDSS alloy in the AR state. Figure 2b–g show the maps for the dispersion of the main alloying elements within the SDSS structure (chromium, nickel, molybdenum, manganese, silicon, and copper). Two main constituent phases are observed after analyzing the distribution maps: a first one rich in Cr (Figure 2b), Mo (Figure 2d), Mn (Figure 2e), and Si (Figure 2f); and a second one enriched in Ni (Figure 2c) and Cu (Figure 2g). Table 1 shows the computed global chemical composition. It can be observed that the weight percentage of alloying elements are consistent with the intervals stated in the main standards (UNS S32750, ASTM A479 F53, AISI F53, WS 1.4410: Cr 24–26%wt; Ni 6–8%wt; Mo 3–5%wt; Mn max. 1.2%wt; Si max. 0.8%wt; Cu max. 0.5%wt; N 0.2–0.3%wt; S max. 0.01%wt; P max. 0.035%wt; C max. 0.03%wt). The presence of oxygen, nitrogen, carbon, and some other elements with a low Z (atomic number) were not quantified, considering the limitations of the SEM-EDS technique.

**Figure 3:**
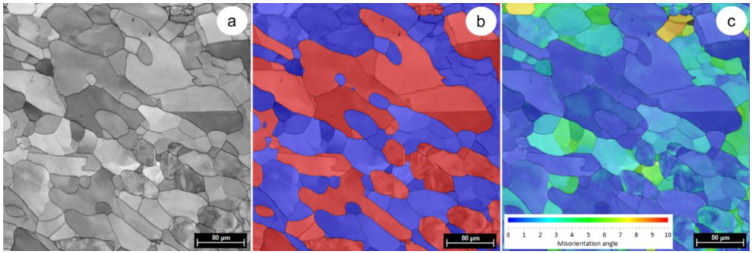
Typical SEM-EBSD microstructure images of AR UNS S32750 Super-Duplex Stainless Steel (**a**); distribution map of constituent phases (γ-phase—red colorized and δ-phase—blue colorized) (**b**); GROD distribution map for both γ-phase and δ-phase (**c**).

**Figure 4:**
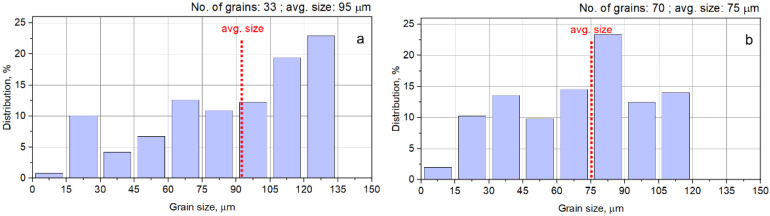
Grain size distribution of γ-phase in AR UNS S32750 Super-Duplex Stainless Steel (**a**); grain size distribution of δ-phase (**b**).

Figure 3a illustrates typical SEM-EBSD microstructural images of the AR UNS S32750 Super-Duplex Stainless Steel. Microstructural analysis showed that the UNS S32750 microstructure in the initial state, is homogeneous, with just two phases being identified (see Figure 3b): (1) austenite-γ, colorized in red, with elongated irregular grains dispersed within the ferrite phase; (2) ferrite-δ colorized in blue, which acts as a metallic matrix. The average grain size of austenite γ is higher than that of ferrite δ, around 95 µm compared to 75 µm—see the grain size distribution from Figure 4. The proportion of constituent phases is approximately 50–52% for δ ferrite and 50–48% for γ austenite. Other secondary phases were not detected. The average chemical composition of both phases (γ and δ) is presented in Table 1.

Grain Reference Orientation Deviation (GROD) distribution map for both γ-phase and δ-phase, in the investigated field, is shown in Figure 3c. The GROD map serves as a tool used for assessing the accumulated deformation or strain at a microstructural level [24,25]; the MO (misorientation) between a reference point and some other points of the considered grain is the basis of GROD. The average orientation of the considered grain was established as a reference point [26,27]. The GROD distribution map of the as-received (AR) UNS S32750 SDSS (Figure 3c) shows that both γ-phase and δ-phase present low stressed grains, with a maximum GROD of 9° recorded for the δ-phase. It can also be observed that the γ-phase shows a lower and more uniform distribution of GROD compared to the δ-phase (Figure 3c). Due to the low GROD, one can assume that the AR UNS S32750 Super-Duplex Stainless Steel shows a low susceptibility to the generation of microcracks.

### 3.2. SEM-EBSD Microstructural Analysis of the Hot-Deformed Alloy

**Figure 5:**
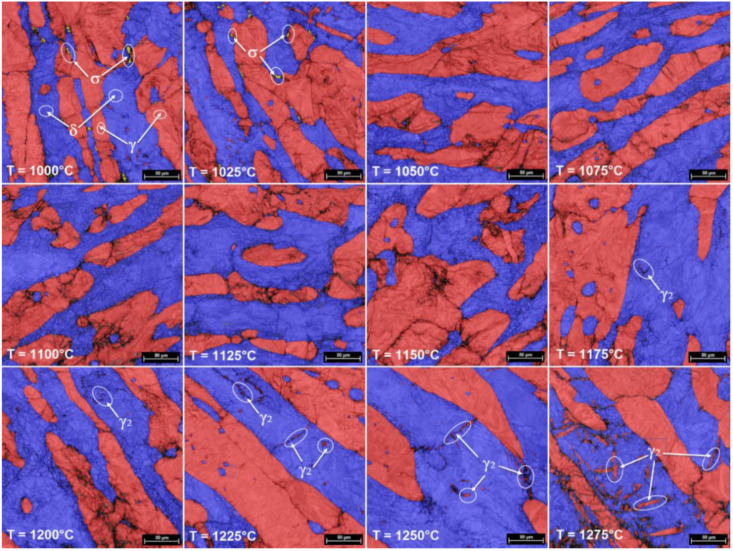
SEM-EBSD images showing typical constituent phase distribution maps in hot-deformed UNS S32750 SDSS alloy (from 1000 °C to 1275 °C).

Figure 5 shows a series of representative SEM-EBSD images for the samples processed by hot upsetting, from 1000 °C to 1275 °C, with a total deformation degree of 30%. For characterizing the microstructural evolution during hot deformation, the following characteristics were analyzed: distribution of constituent phases; the shape and size of grains; GROD distribution map for constituent phases; the occurrence of recrystallization (RX) in δ-phase grains, as well as the secondary austenite phase (γ_2_-phase) precipitation.

The analysis of microstructure evolution, from 1000 °C to 1275 °C, showed the presence of the following constituent phases: ferrite (δ-phase)—blue colorized, primary austenite (γ-phase), and secondary austenite (γ_2_-phase)—red color, and σ-phase—yellow color. At temperatures below 1050 °C, the presence of deleterious σ-phase can be observed, mainly at the δ/γ interface, the decreased quantity of σ-phase fraction, indicating that the dissolution of σ-phase is completed at 1050 °C. Another important observation can be made for temperatures above 1175 °C, where one can observe the presence of a secondary austenite phase (γ_2_-phase), mainly within the δ-phase at the δ/δ interface. This secondary austenite (γ_2_-phase) is generated during heating within an intensely deformed δ-phase matrix by heterogeneous nucleation, in sections where the supersaturation in N of the δ-phase is supporting the precipitation phenomenon. Furthermore, the microstructure inclusions are working as preferential nucleation sites for the γ_2_-phase, which can easily nucleate next to these inclusions [28,29].

**Figure 6:**
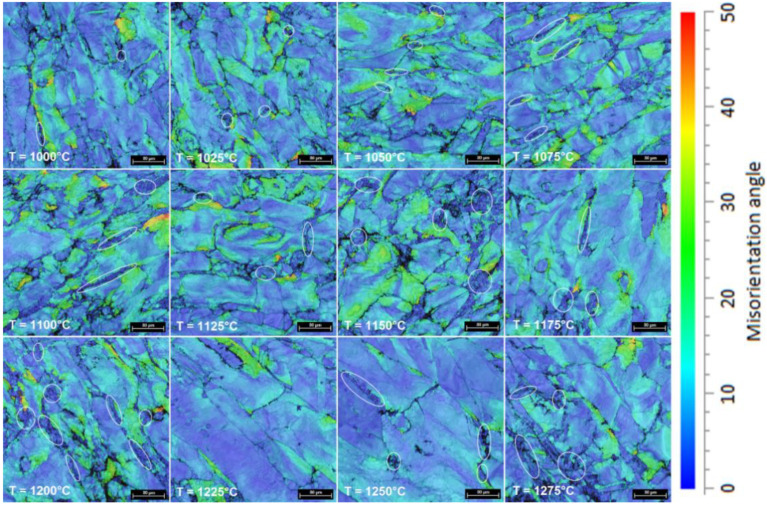
Typical GROD distribution maps, for both γ-phase and δ-phase, in hot-deformed UNS S32750 SDSS alloy (from 1000 °C to 1275 °C).

Figure 6 shows a series of representative GROD distribution maps of samples processed by upsetting, from 1000 °C to 1275 °C, with a total deformation degree of 30%. Analyzing the GROD evolution, one can identify the following: for temperatures between 1000 °C to 1175 °C, the maximum GROD is registered for the δ-phase; while for temperatures between 1200 °C to 1275 °C the maximum GROD is registered for the γ-phase. Analyzing the GROD evolution in the case of δ-phase, it was observed that the GROD increases from 38° (at 1000 °C) to 49° (at 1100 °C), when maximum GROD is recorded, followed by a continuous decreasing to 21° (until 1275 °C). Analyzing the GROD evolution in the case of the γ-phase it was observed that the GROD increases from 26° (at 1000 °C) to 44° (at 1200 °C), followed by a continuous decreasing to 32° (until 1275 °C). It can also be observed that within the δ-phase areas, where GROD shows low-values (marked with white circles); this indicates the recrystallization (RX) occurrence in δ-phase grains.

During deformation of the alloy, the microstructure suffers an increase in defect density, mainly in dislocation density with some new dislocations that are nucleating continuously, from the primary Frank–Read sources. These are blocked at grain level, thus resulting in an increased defect density. Moreover, during hot deformation, the grains suffer rotations to accommodate the increased strain–stress fields. All those effects result in large elastic strains and residual stress fields, visualized as high GROD areas (see Figure 6). The observed behavior, in terms of GROD evolution, suggests that both the stress relieving phenomena and the dynamic recrystallization of new grains phenomena must be considered. The stress relieving phenomena, which occurs during heating, induces important changes within the deformed microstructure of the alloy, decreasing the imperfections density and lowering the residual stress fields and the elastic strains [30,31,32,33]. One must also consider the influence of the dynamic recrystallization of new grains, which can also decrease the elastic strains and residual stress fields [30].

**Figure 7:**
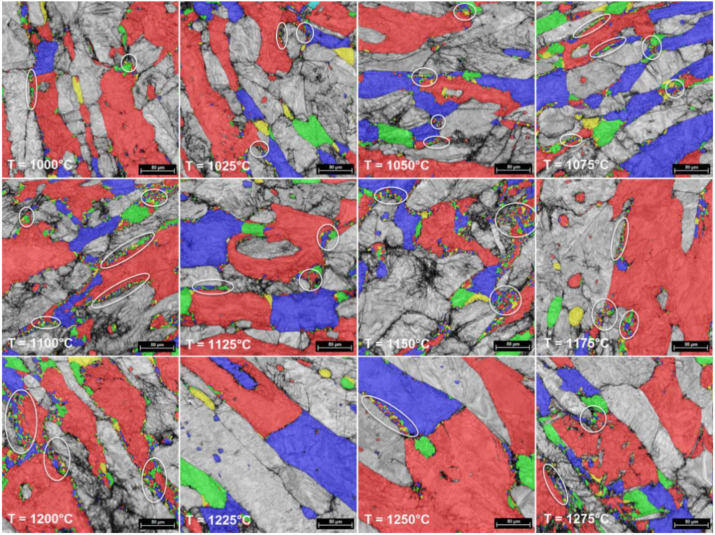
Typical random-colorized δ-phase grain size distribution maps in hot-deformed UNS S32750 SDSS alloy (from 1000 °C to 1275 °C).

**Figure 8:**
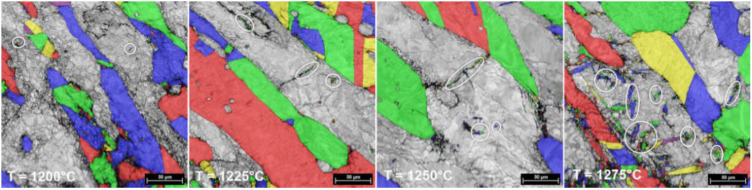
Typical random-colorized γ-phase grain size distribution maps in hot-deformed UNS S32750 SDSS alloy (from 1200 °C to 1275 °C).

By analyzing all the microstructural images, one can observe that in all cases both δ and γ phases show some representative morphologies of strain-hardened microstructures. It is only in the case of δ-phase that some new RX grains are noticed (see Figure 6 and Figure 7). The small size of new RX grains is due to short duration of the hot upsetting process at temperatures ranging from 1000 °C to 1275 °C. By analyzing the influence of the deformation temperature on microstructural evolution, one can observe that the increase in deformation temperature is leading to increased fragmentation in both γ and δ phases, which results in a continuously decreasing average grain size for both phases (see Figure 7 and Figure 8). At 1000 °C, one can observe the presence of some small new ferrite grains, with a low GROD, which shows the occurrence of the RX phenomenon in δ phase (the sectors indicated by white circles from Figure 6 and Figure 7). For deformation temperatures up to 1200 °C, one can observe that the RX mechanism intensifies with the increasing of deformation temperature, which considerably increases the weight fraction of the new recrystallized grains of δ phase (see the sectors pointed by white circles from Figure 7). For the γ phase, no RX phenomena was observed. Moreover, for upsetting temperatures above 1200 °C, the presence of a secondary austenite phase (γ_2_-phase) can be noticed, mainly within the δ-phase at the δ/δ interface, due to the δ → γ phase transition, showing an increased weight fraction with the increase in deformation temperature (Figure 8).

**Figure 9:**
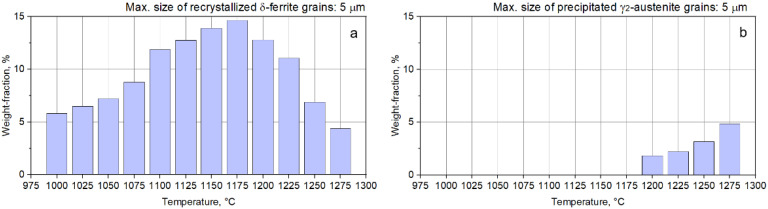
Weight fraction of recrystallized δ-phase (**a**) and precipitated γ_2_-phase (**b**) grains as a function of deformation temperature.

If one considers that all new recrystallized grains show a grain size below 5 µm, then the weight fraction of the recrystallized grains belonging to both the δ-phase and γ_2_-phase can be computed for each deformation temperature. Figure 9 shows the computed weight fraction of the recrystallized δ-phase (a) and precipitated γ_2_-phase (b) grains as a function of deformation temperature. One can observe that, in the case of δ-phase, the weight fraction of recrystallized grains is continuously increasing from 1000 °C (when the recorded weight fraction was close to 5.8%), up to 1175 °C (when the weight fraction is reaching the maximum value, close to 14.6%). Further increasing the upsetting temperature above 1200 °C leads to a decrease in the weight fraction of the δ-phase, with a value close to 4.4% being recorded at 1275 °C. Considering the case of γ_2_-phase, it can be observed that the weight fraction of newly precipitated γ_2_-phase grains is continuously increasing in the 1200–1275 °C range, from 1.8% to 4.9%, due to the δ → γ phase transition, which occurs mainly within the δ-phase at the δ/δ interface (Figure 8).

All the above analysis concerning the microstructural modifications during hot deformation of the experimented material should be correlated with the mechanisms of deformation that govern thermomechanical processing. Since the material contains two phases with different deformation behaviors, the descriptions should be initiated separately on each phase, and then correlated with each other.

When considering the austenite-γ phase, it must be highlighted that it is more ductile than ferrite δ, due to the fcc crystalline structure of γ, as opposed to the bcc crystalline structure of δ. The reason is that the atomic density is nearly double for the fcc crystal structure when compared to the bcc crystal structure [34,35]; this leads to a lower critical energy necessary for activating the slip/twinning processes that assure easier deformation behavior. Consequently, it results that the δ phase (ferrite) manifests a higher strength and resistance to plastic deformation compared to austenite, due to a lower potential to accommodate plastic deformation and a higher critical energy for activating the slip/twinning systems [24,25,26,27].

Taking into account the criteria of minimum activation energy, the easiest slip system to activate for the fcc crystals is the {111} <110> primary system, while for bcc crystals it is the {110} <111> system; as for the easiest to activate twinning system, for the fcc crystals it is the {111} <112> primary system, while for the bcc crystals it is the {112} <111> system [24,25,26,27,35]. Therefore, the double atomic density mentioned above for austenite-γ is manifested in the fcc {111} atomic twinning/slip planes compared to {110} and {112} twinning/slip planes corresponding to the bcc system. As a result, it is understandable why fcc crystalline phases adapt faster to deformation processes than the bcc crystalline phases for the same processing conditions or for the same level of external stress. Additionally, it can be mentioned that, if the deformations of the material are more intense, secondary twinning/slip systems can be activated alongside primary twinning/slip systems, with lower atomic density and higher Miller indices than primary ones [26,27].

## 4. Conclusions

The main results of this research can be summarized as follows:

(a) For the experimented temperature range (1000–1025 °C) applied for hot deforming the UNS S32750 Super-Duplex Stainless Steel by upsetting with a total degree of deformation of 30%, the microstructure of the studied material is composed of approximately equal ratios of γ-phase and δ-phase before and after the hot deforming process.
(b) After all the applied variants of hot deforming, both δ and γ phases showed typical morphologies of strain-hardened structures. During all experimented variants, lateral fissures or cracks were not observed on the surface of UNS S32750 SDSS samples.
(c) The microstructural analysis via SEM-EBSD showed the presence of σ-phase between 1000–1025 °C, at the δ/γ interface; at temperatures above 1050 °C, this deleterious phase was not present due to its complete dissolution.
(d) For the temperature range 1200–1275 °C, the SEM-EBDS analysis indicated the increasingly intense formation of the secondary phase-γ_2_ at the δ/δ interface, as the temperature increased up to 1275 °C. This signaled precipitation process can be correlated with GROD analysis, which indicated a decrease in values for δ in this temperature range, from 49° to 21° i.e., a decreasing stress for δ grains to values that favor the precipitation of γ_2_ as well as intensifying dynamic recrystallization. The small size of the new δ recrystallized grains occurs due to the short duration of the hot deformation process. For the γ phase, no RX mechanism was observed.
(e) Considering the experimented temperatures for hot deforming (1000–1275 °C) and the signalized presence of the deleterious σ-phase between 1000–1025 °C, it can be concluded that the UNS S32750 Super-Duplex Stainless Steel can be safely deformed by upsetting between 1050–1275 °C with an experimented total degree of deformation of 30%.

## Figures and Tables

**Table 1:** The average chemical composition of the AR UNS S32750 Super-Duplex Stainless Steel.

Constituent Phase	Chemical Composition, [%, wt]						
	Cr	Ni	Mo	Mn	Si	Cu	Fe
*global*	25.85 ± 0.10	6.62 ± 0.12	3.05 ± 0.11	0.46 ± 0.09	0.38 ± 0.05	0.19 ± 0.03	balance
δ-*phase*	28.42 ± 0.09	5.17 ± 0.04	3.73 ± 0.12	0.48 ± 0.01	0.39 ± 0.01	0.15 ± 0.01	balance
γ-*phase*	25.28 ± 0.04	8.06 ± 0.04	2.31 ± 0.01	0.42 ± 0.02	0.37 ± 0.01	0.23 ± 0.01	balance

## Data Availability

The data presented in this study are available on request from the corresponding author.

## References

[1] Xiong J., Tan M.Y., Forsyth M. (2013). The corrosion behaviors of stainless steel weldments in sodium chloride solution observed using a novel electrochemical measurement approach. Desalination.

[2] Zanotto F., Grassi V., Balbo A., Zucchi F., Monticelli C. (2019). Investigation on the Corrosion Behavior of Lean Duplex Stainless Steel 2404 after Aging within the 650–850 °C Temperature Range. Metals.

[3] Zanotto F., Grassi V., Balbo A., Monticelli C., Zucchi F. (2014). Stress corrosion cracking of LDX 2101^®^ duplex stainless steel in chloride solutions in the presence of thiosulphate. Corros. Sci..

[4] Tavares S.S.M., Silva V.G., Pardal J.M., Corte J.S. (2013). Investigation of stress corrosion cracks in a UNS S32750 superduplex stainless steel. Eng. Fail. Anal..

[5] Nilsson J.O. (1992). Super Duplex Stainless Steels. J. Mater. Sci. Technol..

[6] Hoseinpoor M., Momeni M., Moayed M.H., Davoodi A. (2014). EIS assessment of critical pitting temperature of 2205 duplex stainless steel in acidified ferric chloride solution. Corros. Sci..

[7] Fargas G., Anglada M., Mateo A. (2009). Effect of the annealing temperature on the mechanical properties, formability and corrosion resistance of hot-rolled duplex stainless steels. J. Mater. Process. Technol..

[8] Maki T., Furuhara T., Tsuzaki K. (2001). Microstructure Development by Thermomechanical Processing in Duplex Stainless Steel. ISIJ Int..

[9] Moverare J.J., Odén M. (2002). Deformation behaviour of a prestrained duplex stainless steel. Mater. Sci. Eng. A.

[10] Kleber S., Hafok M. (2010). Multiaxial Forging of Super Duplex Steel. Mat. Sci. Forum.

[11] Rys J., Cempura G. (2017). Microstructure and deformation behavior of metastable duplex stainless steel at high rolling reductions. Mater. Sci. Eng. A.

[12] Primig S., Ragger K.S., Buchmayr B. (2013). EBSD Study of the Microstructural Evolution during Hot Compression Testing of a Superduplex Steel. Mat. Sci. Forum..

[13] Kim S.K., Kang K.Y., Kim M.S., Lee J.M. (2015). Low-Temperature Mechanical Behavior of Super Duplex Stainless Steel with Sigma Precipitation. Metals.

[14] Pettersson N., Wessman S., Thuvander M., Hedström P., Odqvist J., Pettersson R.F.A., Hertzman S. (2015). Nanostructure evolution and mechanical property changes during aging of a super duplex stainless steel at 300 °C. Mater. Sci. Eng. A.

[15] Liu G., Wang Y., Li S., Du K., Wang X. (2016). Deformation behavior of thermal aged duplex stainless steels studied by nanoindentation, EBSD and TEM. Mater. High. Temp..

[16] Wroński S., Tarasiuk J., Bacroix B., Baczmański A., Braham C. (2012). Investigation of plastic deformation heterogeneities in duplexsteel by EBSD. Mater. Charact..

[17] Dakhlaoui R., Baczmański A., Braham C., Wroński S., Wierzbanowski K., Oliver E.C. (2006). Effect of residual stresses onindividual phase mechanical properties of austeno-ferriticduplex stainless steel. Acta Mater..

[18] Kang J.H., Heo S.J., Yoo J., Kwon Y.C. (2019). Hot working characteristics of S32760 super duplex stainless steel. J. Mech. Sci. Technol..

[19] Cojocaru V.D., Serban N., Angelescu M.L., Cotrut M.C., Cojocaru E.M., Vintila A.N. (2017). Influence of Solution Treatment Temperature on Microstructural Properties of an Industrially Forged UNS S32750/1.4410/F53 Super Duplex Stainless Steel (SDSS) Alloy. Metals.

[20] Cojocaru V.D., Raducanu D., Angelescu M.L., Vintila A.N., Serban N., Dan I., Cojocaru E.M., Cinca I. (2017). Influence of Solution Treatment Duration on Microstructural Features of an Industrial Forged UNS S32750/1.4410/F53 Super Duplex Stainless Steel (SDSS) Alloy. JOM.

[21] Örnek C., Engelberg D.L. (2016). Towards understanding the effect of deformation mode on stress corrosion cracking susceptibility of grade 2205 duplex stainless steel. Mater. Sci. Eng. A.

[22] Serban N., Cojocaru V.D., Angelescu M.L., Raducanu D., Cinca I., Vintila A.N., Cojocaru E.M. (2019). High temperature deformation behaviour of an industrial S32760/1.4501/F55 super duplex stainless steel (SDSS) alloy. Metall. Ital..

[23] Angelescu M.L., Cojocaru V.D., Serban N., Cojocaru E.M. (2018). Evaluation of Optimal Forging Temperature Range for an Industrial UNS S32750 SDSS Alloy Using SEM-EBSD Analysis. Metals.

[24] Schayes C., Bouquerel J., Vogt J.B., Palleschi F., Zaefferer S. (2016). A comparison of EBSD based strain indicators for the study of Fe-3Si steel subjected to cyclic loading. Mater. Charact..

[25] Kamaya M. (2009). Characterization of microstructural damage due to low-cycle-fatigue by EBSD observation. Mater. Charact..

[26] Wright S.I., Nowell M.M., Field D.P. (2011). A review of strain analysis using electron backscatter diffraction. Microsc. Microanal..

[27] Kamaya M. (2012). Assessment of local deformation using EBSD: Quantification of local damage at grain boundaries. Mater. Charact..

[28] Magalhaes C.H.X.M., Faria G.L., Lagoeiro L.E., Silva J.D. (2017). Characterization of the austenite reformation mechanisms as a function of the Initial ferritic state in a UNS S32304 duplex stainless steel. Mater. Res..

[29] Muthupandi V., Srinivasan P.B., Shankar V., Seshadri S.K., Sundaresan S. (2005). Effect of nickel and nitrogen addition on the microstructure and mechanical properties of power beam processed duplex stainless steel (UNS 31803) weld metals. Mater. Lett..

[30] Sun Z.Q., Yang W.Y., Qi J.J., Hu A.M. (2002). Deformation enhanced transformation and dynamic recrystallization of ferrite in a low carbon steel during multipass hot deformation. Mater. Sci. Eng. A.

[31] Ciuffini A.F., Barella S., Peral Martínez L.B., Mapelli C., Fernández Pariente I. (2018). Influence of Microstructure and Shot Peening Treatment on Corrosion Resistance of AISI F55-UNS S32760 Super Duplex Stainless Steel. Materials.

[32] Mészáros I., Bögre B. (2019). Complex Study of Eutectoidal Phase Transformation of 2507-Type Super-Duplex Stainless Steel. Materials.

[33] Biserova-Tahchieva A., Cabrera J.M., Llorca-Isern N. (2020). Study of the Thermochemical Surface Treatment Effect on the Phase Precipitation and Degradation Behaviour of DSS and SDSS. Materials.

[34] Liang Z.Y., Huang M.X. (2015). Deformation twinning in small-sizedface-centred cubic single crystals: Experiments and modelling. J. Mech. Phys. Solids.

[35] Chen Z., Cai H., Li S., Zhang X., Wang F., Tan C. (2007). Analysis ofcrystallographic twinning and slip in fcc crystals underplane strain compression. Mater. Sci. Eng. A.

